# Multiomics analysis of human peripheral blood reveals marked molecular profiling changes caused by one night of sleep deprivation

**DOI:** 10.1002/mco2.252

**Published:** 2023-04-30

**Authors:** Chongyang Chen, Jing Wang, Chao Yang, Haitao Yu, Bingge Zhang, Xiao Yang, Bocheng Xiong, Yongmei Xie, Shupeng Li, Zaijun Zhang, Feiqi Zhu, Jianjun Liu, Gong‐Ping Liu, Xifei Yang

**Affiliations:** ^1^ Shenzhen Key Laboratory of Modern Toxicology, Shenzhen Medical Key Discipline of Health Toxicology (2020‐2024) Shenzhen Center for Disease Control and Prevention Shenzhen China; ^2^ Key Laboratory of Nuclear Medicine, Ministry of Health, Jiangsu Key Laboratory of Molecular Nuclear Medicine Jiangsu Institute of Nuclear Medicine Wuxi China; ^3^ Cognitive Impairment Ward of Neurology Department The 3rd Affiliated Hospital of Shenzhen University Shenzhen China; ^4^ Department of Pathophysiology, School of Basic Medicine and the Collaborative Innovation Center for Brain Science, Key Laboratory of Ministry of Education of China and Hubei Province for Neurological Disorders, Tongji Medical College Huazhong University of Science and Technology Wuhan China; ^5^ State Key Laboratory of Biotherapy and Cancer Center, West China Hospital Sichuan University and Collaborative Innovation Center of Biotherapy Chengdu China; ^6^ School of Chemical Biology and Biotechnology Peking University Shenzhen Graduate School Shenzhen China; ^7^ Institute of New Drug Research and Guangzhou, Key Laboratory of Innovative Chemical Drug Research in Cardio‐cerebrovascular Diseases Jinan University College of Pharmacy Guangzhou China; ^8^ Co‐innovation Center of Neurodegeneration Nantong University Nantong China

**Keywords:** immune disorder, melatonin, multiomics, neurodegenerative disease, neutrophils, sleep deprivation

## Abstract

Sleep insufficiency is associated with various disorders; the molecular basis is unknown until now. Here, 14 males and 18 females were subjected to short‐term (24 h) sleep deprivation, and donated fasting blood samples prior to (day 1) and following (days 2 and 3) short‐term sleep deprivation. We used multiple omics techniques to examine changes in volunteers’ blood samples that were subjected to integrated, biochemical, transcriptomic, proteomic, and metabolomic analyses. Sleep deprivation caused marked molecular changes (46.4% transcript genes, 59.3% proteins, and 55.6% metabolites) that incompletely reversed by day 3. The immune system in particular neutrophil‐mediated processes associated with plasma superoxidase dismutase‐1 and S100A8 gene expression was markedly affected. Sleep deprivation decreased melatonin levels and increased immune cells, inflammatory factors and c‐reactive protein. By disease enrichment analysis, sleep deprivation induced signaling pathways for schizophrenia and neurodegenerative diseases enriched. In sum, this is the first multiomics approach to show that sleep deprivation causes prominent immune changes in humans, and clearly identified potential immune biomarkers associated with sleep deprivation. This study indicated that the blood profile following sleep disruption, such as may occur among shift workers, may induce immune and central nervous system dysfunction.

## Graphical abstract

We firstly reported multiomics of peripheral blood after sleep deprivation SOD1 and S100A8 may serve as biomarkers for sleep deprivation caused immune disorder Brief sleep recovery cannot repair the harmful effects of sleep deprivation Melatonin potentially linked to sleep deprivation‐induced neurodegenerative diseases:

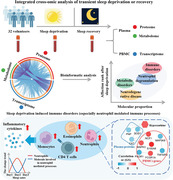

## 1 INTRODUCTION

Circadian rhythms are closely related to biological activity and health, and the health hazards of insufficient sleep or circadian rhythm disorders are of increasing concern.^1^ Several studies have shown that insufficient sleep (usually less than 6 h/day) is associated with various health disorders, such as cardiovascular disease,^2^ diabetes mellitus,^3^ obesity,^4^ cognitive impairment,^5^, ^6^ among others. In addition, short‐ or long‐term insomnia may be a risk factor for stroke and death,^7^ and <6 h sleep/day raised by 20% the risk of myocardial infarction.^8^ Recent research has found that just one night of sleep deprivation increased Aβ amyloid in brain regions.^9^ In sum, long‐term or short‐term lack of sleep or sleep disorder (insomnia) appears to be potential risk factors for various diseases.

Even though there are a lot of studies reported many diseases induced by sleep disorder or deprivation, for example, sleep deprivation induced the accumulation of ROS (reactive oxygen species) in gut resulting animal death,^10^ sleep deficiency was reported to impair molecular clearance from the human brain,^11^ the detail mechanisms are still unclear. Sleep has a crosslink with immunity, immune system activation alters sleep, which in turn affects the nature of innate and adaptive immune systems, and the unbalance between sleep and immunity can lead to disease.^12^ Sleep disorder was found to deteriorate immune functions, and thus induced various diseases, such as infection,^13^ cancer,^14^ neurodegenerative disease,^15^ autoimmune disease,^16^ metabolic, and vascular disease.^17^ However, the current studies mainly focused on changes in the number of immune cells and levels of inflammatory factors, such as neutrophil and T cells,^18^ c‐reactive protein (CRP), and IL‐6,^19^ etc. In order to provide the prevention guidance for sleep insufficiency related diseases, there is a need to further explore the molecular basis of immune changes caused by insufficient sleep. The development of high throughput technology (genome, transcriptome, proteome, and metabolome) has provided a potential possibility. At present, there has little omics studies in sleep deprivation, and most of them involved only a single omics. Therefore, human‐based multiomics studies will be more helpful to reveal the molecular mechanisms for sleep deprivation‐induced immune disorders and related diseases.

Multiomics analysis of peripheral blood samples provides a window to monitor macroscopic and microenvironmental changes in vivo. The transcriptomics of peripheral whole blood or leukocytes reflects the integrated physiological status of multiple organs.^20^, ^21^ Plasma proteomics of peripheral blood provides a method to monitor disease biomarkers.^22^ Metabolomics is an endpoint indicator of environmental changes in vivo after gene expression and protein translation.^23^, ^24^ Therefore, contemporary assessment of the transcriptome, proteome and metabolome of peripheral blood provides a comprehensive method to assess health status, including adverse changes associated with sleep disorders. Blood transcriptomic changes resulting from chronic sleep deprivation (1 week) have been reported,^25^ as have the characteristics of the plasma metabolome after sleep deprivation (24 h)^26^ and the effect of 24 h of irregular diet and sleep on plasma protein expression.^27^ However, the aforementioned studies employed single (not multiomics) categories to assess the effects of circadian rhythm changes on peripheral blood within 24 h; in addition, the number of participants was small, and some studies were based on a single gender. Thus, there is no comprehensive multiomics analysis that integrates the blood‐based physiological effects of transient sleep deprivation and sleep recovery.

The present study, which employs a multiomics approach to address this question in healthy adults, finds that short‐term (24 h) sleep deprivation perturbs for at least 1−2 days both the immune system and the molecular framework associated with disorders of the nervous system. While the findings suggest that short‐term sleep supplementation cannot compensate for the adverse physiological effects promoted by shift work, they provide biomarkers that correlate with sleep deprivation that may be useful for the prevention of associated diseases.

## 2 RESULTS

### 2.1 Characterization of plasma molecules and physiological changes after transient sleep deprivation

**FIGURE 1:**
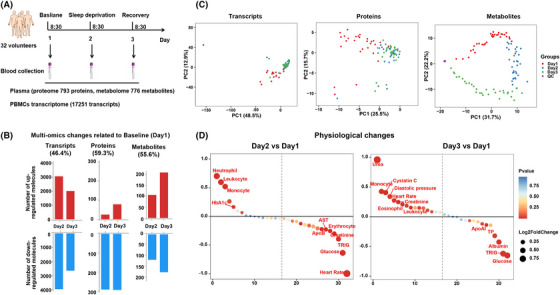
**Transient sleep deprivation induced changes in multiple‐omics molecular and physiological indicators**. (A) Basic procedures for sleep deprivation experiments. (B) The statistical analysis of multiomics molecules on day 2 and day 3 relative to baseline (day 1). (C) Principal component analysis (PCA) revealed changes in the data distribution of genes, proteins and metabolites after sleep deprivation. (D) Difference analysis of physiological indicators (heart rate, blood pressure, blood glucose and lipids, liver and kidney function, and blood routine) after sleep deprivation.

A total 17,251 genes were detected in the primary blood mononuclear cell (PBMC) transcriptome, 793 high‐confidence proteins in the plasma proteome, and 776 metabolites in the plasma metabolome (Figure 1A). The differential analysis showed that approximately half of the plasma molecules (46.4% of genes, 59.3% of plasma proteins, 55.6% of metabolites) changed after 24‐h sleep deprivation; most genes were downregulated and proteins decreased, while the expression of metabolites was slightly increased (Figure 1B). Unsupervised principal component analysis (PCA) found that sleep deprivation discriminated the expression of the transcriptome and proteome between baseline (day 1) and sleep deprivation (day 2) or brief sleep recovery (day 3), while the expression levels between day 2 and day 3 were basically similar. Comparable analysis of metabolome data showed that sleep deprivation resulted in sustained changes in metabolites (Figure 1C). PCA analysis suggested that the brief sleep recovery period (day 3) was actually a continued effect of the period of sleep deprivation (day 2). Figure 1D shows that when compared with baseline data (day 1), sleep deprivation was associated with a significant increase in immune cells (leukocytes, neutrophils, monocytes) and HbA1c, while significant decreases were found for blood glucose concentration, triglyceride (TRIG), ApoB, heart rate, creatinine, aspartate aminotransferase (AST), and erythrocytes (day 1). At day 3, blood glucose, triglyceride, albumin, and total protein were significantly decreased, and blood pressure, heart rate, blood urea, cystatin c, leukocytes, monocytes, and eosinophils were significantly increased (Figure 1D). These results suggested that short‐term recovery after sleep deprivation can rapidly restore heart rate but failed to reverse immune function (immune cells) and metabolic processes (blood glucose, blood lipids, etc.), indicating that sleep deprivation has a longer‐lasting effect on physiological factors related to immunity and metabolism. These data suggested that just one night sleep deprivation induced an enormous effect, which nearly half of the blood molecules changed.

### 2.2 Sleep deprivation resulted in differentially expressed plasma proteins mainly enriched in immune process and metabolic process

The results of plasma proteomics found that, compared with Day1, a total of 316 proteins was changed on day 2. The Volcano plot showed that most of the proteins (*n* = 292) were reduced, and only 24 proteins were increased. Biological process revealed that these differentially expressed (DE) proteins (subsequently referred to as “differential proteins”) were enriched in the functions of neutrophil‐mediated immunity, leukocyte degranulation, myeloid cell activation involved in immune processes, neutrophil degranulation, among others. Pathway analysis found that DE proteins participated in complement and coagulation cascades, antigen processing and presentation, cholesterol metabolism, focal adhesion, phagosome, Parkinson's disease, and glycolysis/gluconeogenesis (Figures S1A and S2A). Compared with Day1, 372 proteins were changed on day 3, of which 78 proteins were increased and 294 proteins decreased. The main biological processes included wound healing, neutrophil‐mediated immunity, neutrophil degranulation, coagulation, complement activation, among others, and pathways focused on complement and coagulation cascade, focal adhesion, phagosome, Parkinson's disease, glycolysis/gluconeogenesis, and estrogen signaling pathway (Figures S1B and S2B). In concert, these results indicated many common changes in the differential protein pattern on day 2 and day 3 after 24 h of sleep deprivation. For example, most proteins showed reduced expression, and the differential proteins were mainly enriched in immune processes (neutrophil activation, complement and coagulation cascades, focal adhesion, phagosome), metabolic processes (glycolysis/gluconeogenesis) and Parkinson's disease, suggesting that sleep deprivation may affect immune, metabolic and brain function. In addition, compared with day 2, the expression of 179 proteins was changed (100 increased and 79 decreased) on day 3; these were involved in humoral immune response, complement activation, acute inflammatory response, platelet degranulation, among others. Complement and coagulation cascade, cholesterol metabolism were the main enriched pathways (Figures S1C and S2C), while the addition of data of day 2 versus day 1 plus day 3 versus day 1, revealed that immune‐related molecules and pathways were extremely sensitive to sleep deprivation and sleep recovery.

To identify biological processes and pathways associated with sleep deprivation and recovery, the time cluster analyzed 470 total differential proteins (summarized DE proteins from day 2 vs. day 1 and day 3 vs. day 1). Differential proteins were divided into Cluster 1 (89 proteins decreased on day 2 and increased on day 3), Cluster 2 (89 proteins decreased on day 3), Cluster 3 (213 proteins decreased on day 2 and day 3), and Cluster 4 (79 proteins increased on day 3). Cluster 1 proteins were mainly involved in neutrophil‐mediated immunity, cholesterol metabolism, complement and coagulation cascade, etc. Cluster 2 proteins were involved in complement activation‐related processes, complement and coagulation, among others. Cluster 3 proteins participated in platelet degranulation and activation, coagulation, carbon metabolism and glycolysis/gluconeogenesis. Cluster 4 proteins were focused on complement activation, immune response‐related processes, and pathways (Figure S3). The cluster analysis showed that neutrophil‐mediated immune processes were consistent with sleep changes, while expression of proteins involved in platelet activation, coagulation, glucose or carbon metabolism proteins remained low after sleep deprivation, while the proteins involved in complement‐related processes showed no regularity.

**FIGURE 2:**
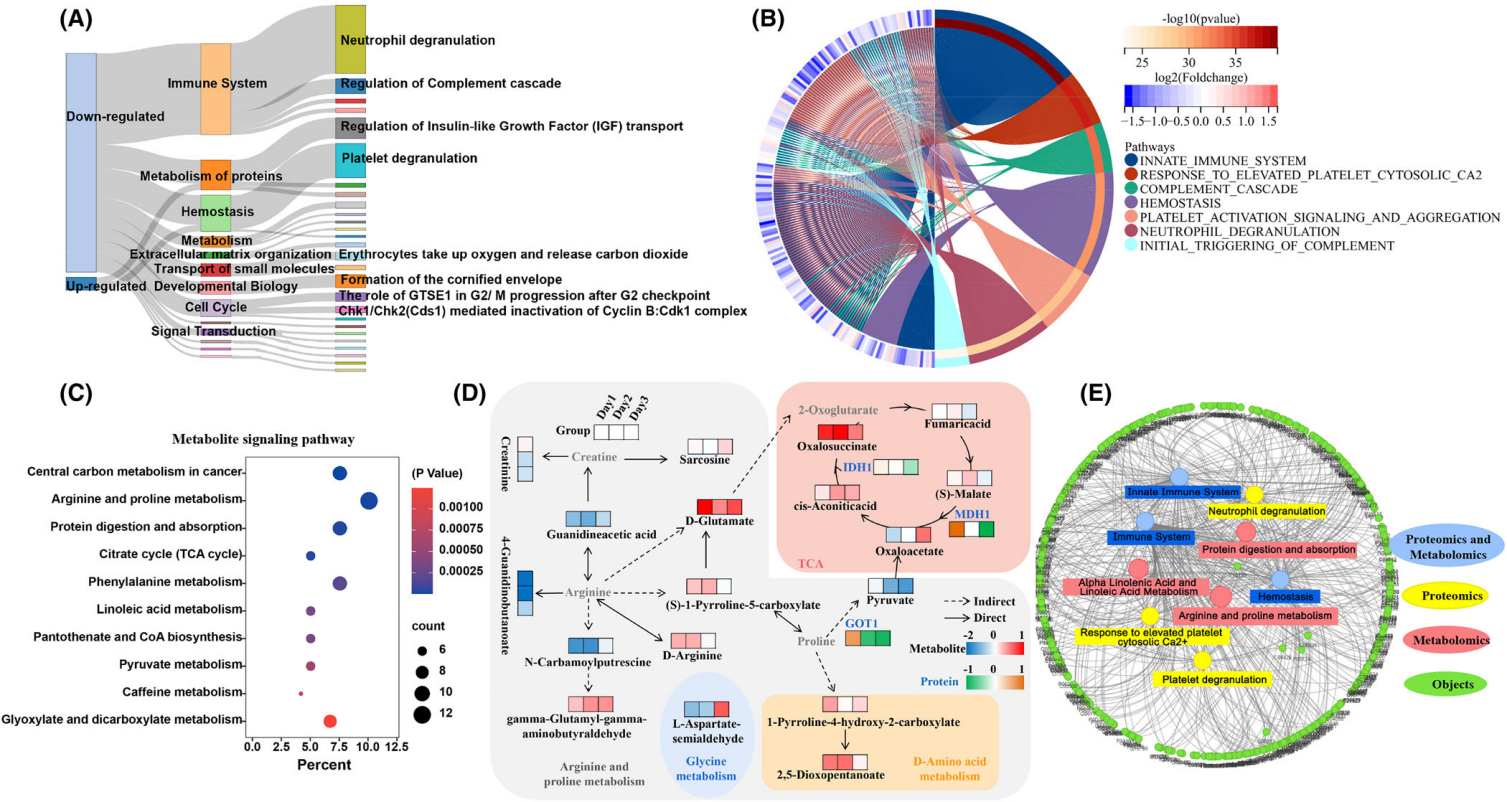
**Proteomics and metabolomics showed significant changes in immune‐related processes after sleep deprivation**. Reactome analysis classified the signaling pathways and biological processes of the total differential proteins after sleep deprivation. (A) The Sankey diagram of signaling pathways. (B) Chord diagram of biological processes. The fold change of total differential proteins was the mean fold change related to baseline (day 1). (C) Metabolic pathway analysis for total differential metabolites in plasma after sleep deprivation; the top 10 pathways are shown. (D) The pathway map of arginine and proline metabolism and TCA cycle after sleep deprivation. The metabolite level is shown as blue to red, that is, from low level to high level. The protein expression is shown as green to orange, that is, from low expression to high expression. (E) Wukong Cloud platform for enrichment analysis of differential metabolites and proteins. Shown are the top three metabolic pathways, protein pathways and pathways of interaction between metabolism and protein.

Reactome Pathway hierarchical enrichment analysis showed that immune‐related signaling pathways ranked first, followed by protein metabolism, hemostasis, metabolism, and other related signaling pathways, among which neutrophil degranulation accounted for the largest proportion in the immune system (Figure 2A). Hierarchical analysis of biological process also showed that the innate immune system accounted for the largest proportion, followed by hemostasis, neutrophil degranulation, complement cascade, among others (Figure 2B). In addition, immunoglobulin continued to be downregulated during sleep deprivation and recovery; complement proteins and cholesterol metabolism proteins were mostly decreased after sleep deprivation and began to increase after sleep recovery, and glycolysis and gluconeogenesis proteins were mostly decreased after sleep deprivation and remained down after sleep recovery (Figure S4). Plasma proteomics showed that immune processes were the main changes after sleep deprivation, followed by metabolic processes. The neutrophil‐mediated immune processes were closely related to the dynamic changes associated with sleep. The above data showed that sleep deprivation induced the prominent enrichment of DE plasma proteins involved in immune‐related biological processes and pathways.

### 2.3 Combined metabolome and proteome analysis revealed the dominance of immune changes after sleep deprivation

Metabolomics found that 223 metabolites were changed on day 2 after sleep deprivation, of which 117 metabolites were decreased and 106 metabolites were increased. Differential metabolites were enriched in central carbon metabolism in cancer, protein degradation and absorption, pyruvate metabolism, arginine, and proline metabolism (Figure S5A). Compared with day 1, 383 metabolites were changed on day 3, among which 209 metabolites were increased, and 174 metabolites were decreased. These metabolites were also enriched in central carbon metabolism in cancer, protein degradation and absorption, arginine and proline metabolism. Additionally, the metabolites of day 3 versus day 2 were still enriched in arginine and proline metabolism, and in pyruvate metabolism (Figure S5B,C). Metabolic classification found that orotic acid (which is involved in nucleotide metabolism) was persistently elevated, while TCA (tricarboxylic acid)‐cycle pyruvate continued to decrease and oxaloacetate continued to increase after sleep deprivation. Most metabolites in lipid metabolism decreased after sleep deprivation, especially 3‐hydroxybutanoate and linoleic acid. Moreover, amino acids accounted for a large proportion of metabolites, and levels of D‐glutamate, phenylacetaldehyde, guanidinoacetic acid, hippuric acid synchronized with sleep changes (Figure S6).

Searching the KEGG (kyoto encyclopedia of genes and genomes) compound database revealed a total 298 differential metabolites after sleep deprivation, with enriched pathways mainly in arginine and proline metabolism, followed by carbon metabolism‐related processes (Figure 2C). The map of arginine and proline metabolism and of the TCA cycle showed that most metabolites were decreased after sleep deprivation; these included D‐glutamate, pyruvate, guanidinoacetic acid, and creatinine, among others, and the pathway‐involved proteins (IDH1, MDH1, GOT1) were also decreased (Figure 2D), while arginine and proline metabolism were closely related to the immune process. This result indicated that sleep deprivation was associated with some degree of immunosuppression, a result consistent with proteomic data showing of the reduced presence of most proteins involved in immune pathways. In addition, interaction analysis of differential proteins and metabolites showed that the top 3 pathways for protein‐metabolic interaction were innate immune system, immune system and hemostasis (Figure 2E). Analysis of protein‐metabolism interaction showed that immune‐related processes still dominated the changes induced by sleep deprivation, and arginine and proline metabolism were the main metabolic processes, followed by TCA cycle‐related metabolic processes. Integrative metabolomic and proteomic analyses suggested that immune function changes are the most prominent response to sleep deprivation.

### 2.4 Transcriptome and proteome correlation analysis highlighted neutrophil‐mediated immune disorder

**FIGURE 3:**
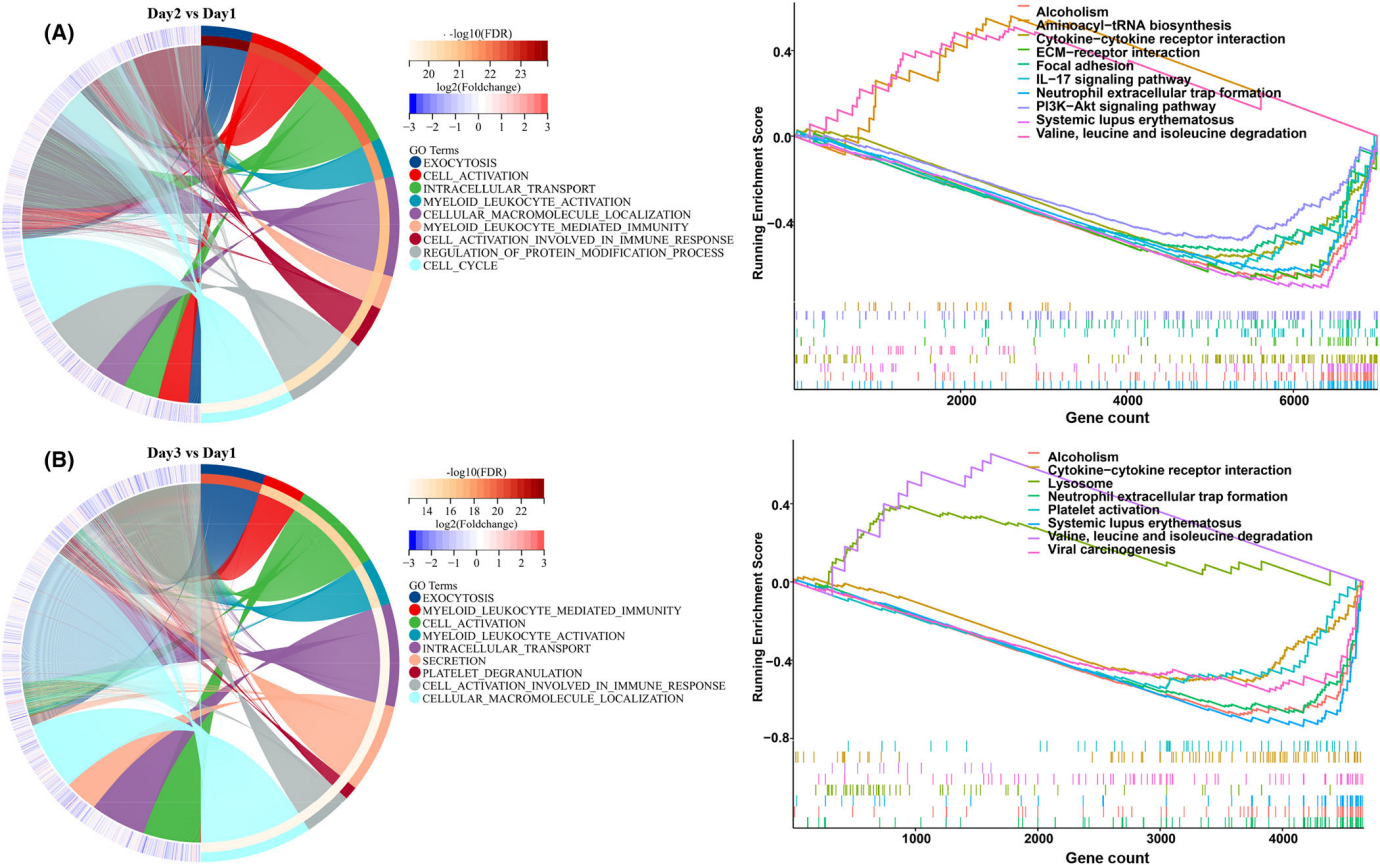
**The transcriptome of peripheral blood mononuclear cell (PBMCs) after sleep deprivation**. Chord diagram showing enriched biological processes associated with differentially expressed genes, and the results of gene set enrichment analysis (GSEA) to identify potential signaling pathways after sleep deprivation. (A) Chord diagram and GSEA analysis of differential genes on day 2 and (B) day 3. The *p* value of enriched biological processes and pathways were corrected by Bonferroni‐Holm (BH).

Since proteomic and metabolomic data revealed that immune changes were significantly altered after sleep deprivation, we performed transcriptomic analysis of PBMCs to find the core genes that induced observed changes in measures of immune function. Among 17,251 genes, 7998 genes (Q < 0.05) were changed on day 2 and day 3 after sleep deprivation. Among them, 6498 genes with FPKM ≥ 1, and 625 genes with fold change (FC) ≥2. Compared with day 1, the expression of 3063 genes was upregulated and 3984 genes downregulated after sleep deprivation (day 2). The biological processes of DE genes were enriched in exocytosis, cell activation, intracellular transport, myeloid leukocyte activation. Gene set enrichment analysis (GSEA) identified the suppressed signaling pathways were: cytokines‐cytokine receptor interaction, cell adhesion, IL‐17 signaling pathway, and neutrophil extracellular trap formation, among others, and the activated signaling pathways included aminoacyl−tRNA biosynthesis, valine, leucine and isoleucine degradation (Figure 3A). On day 3, 2019 genes were upregulated and 2663 genes were downregulated, and biological process focused mainly on exocytosis, myeloid leukocyte‐mediated immunity, cell activation, platelet degranulation, among others. The GSEA showed that the activation signal pathways included lysosome, valine, leucine, and isoleucine degradation, while the suppressed signal pathways included cytokine‐cytokine receptor interaction, neutrophil extracellular trap formation, among others (Figure 3B). Molecular Complex Detection analysis was used to find the core genes regulated after sleep deprivation. For day 2 versus day 1, the DE genes were mainly enriched in chemokines and inflammatory responses, immune and inflammatory responses, platelet degranulation and neutrophil degranulation, and oxygen transport (Figure S7A). DE genes on Day 3 versus day 1 included those involved in immune and inflammatory responses, oxygen transport and leukocyte migration, and platelet degranulation (Figure S7B). In addition, the core DE genes on day 2 and day 3 were basically downregulated, which was consistent with the reduction of most proteins in the plasma proteome. Additionally, the genes and proteins associated with immune processes showed, respectively, a mostly reduced expression and presence after sleep deprivation.

**FIGURE 4:**
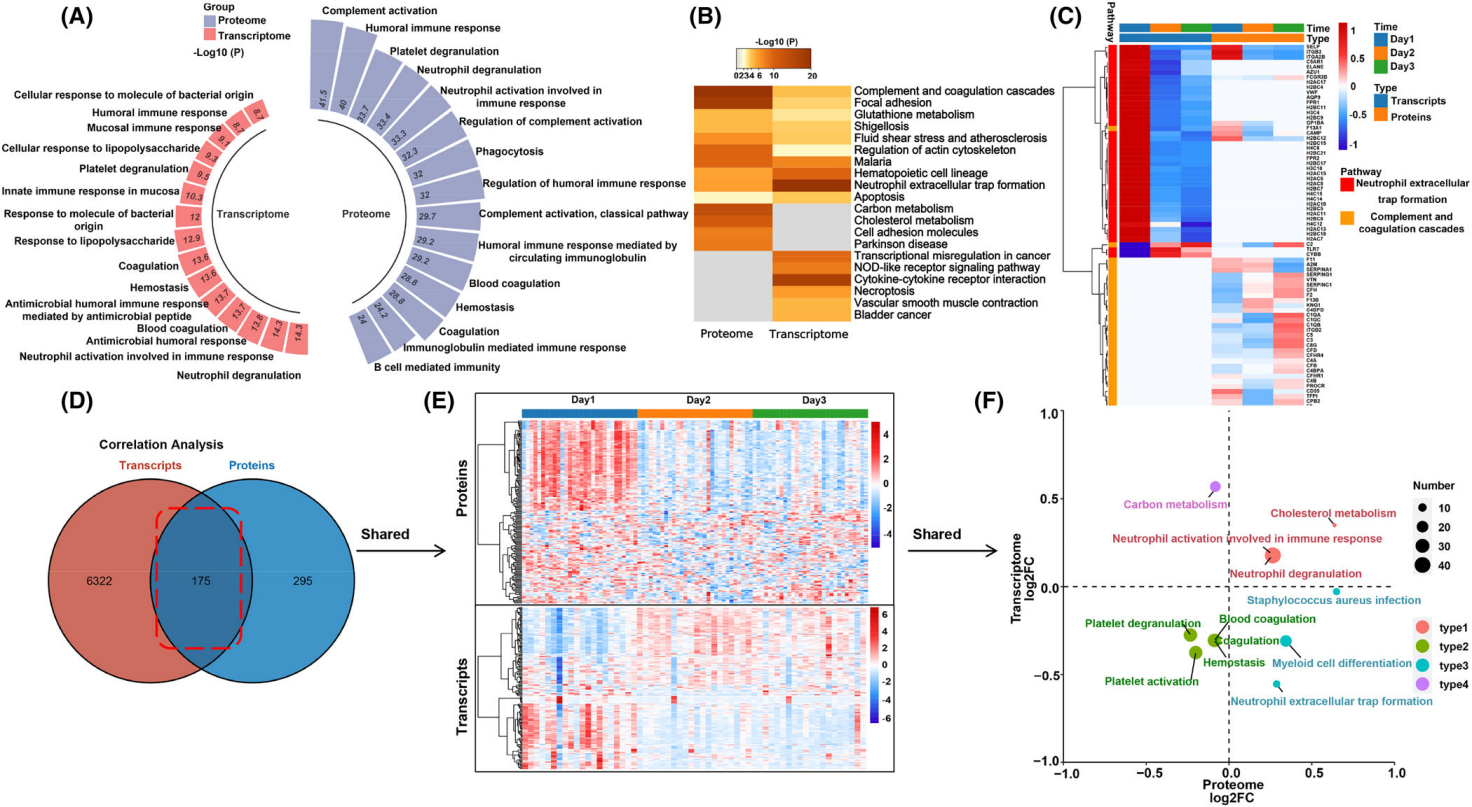
**Correction analysis of differential genes and proteins after sleep deprivation**. (A) GO functional analysis of total differential genes (FC ≥ 2) and proteins after sleep deprivation. Top 15 biological processes are shown. (B) KEGG pathway analysis of differential genes and proteins. The darker the color, the smaller the *p* value. (C) Heat map analysis of differential genes and proteins in pathways of neutrophil extracellular trap formation, complement and coagulation cascade. Blue represents low expression abundance and red represents high expression abundance. (D) Venny analysis of total differentially expressed genes and proteins after sleep deprivation (day 3 or day 2 vs. day 1), and (E) Heat map of shared omics molecules. (F) 2D annotation analysis of the shared differential genes and proteins. The mean fold change of genes or proteins is shown along with the biological processes and pathways with p value less than 0.05.

Since the results of the PBMC transcriptome and plasma proteome were consistent, we subsequently performed correlation analysis to find common gene and protein modules. Biological process analysis found that the differential plasma proteins caused by sleep deprivation were mainly enriched in complement activation, immune response, neutrophil degranulation and immune response, platelet degranulation, complement activation and cascade, among others, and the differential genes (FC ≥ 2) of the transcriptome were also mainly enriched in neutrophil degranulation, neutrophil activation involved in immune response, hemostasis, platelet degranulation, among others (Figure 4A). KEGG pathway analysis showed that neutrophil extracellular trap formation, complement and coagulation cascades with the smallest *p* value were covered in the enrichment of differential genes and differential proteins (Figure 4B). Moreover, the heat map of differential genes and proteins from these two pathways showed that most genes in the neutrophil extracellular trap formation were downregulated on day 2 and day 3 after sleep deprivation, while many proteins in the complement and coagulation cascades were decreased on day 2 and increased on day 3 (Figure 4C). This difference indicated that the variation trend of genes and proteins was different in the two immune system‐related pathways. In addition, the coverage of genes and proteins in neutrophil extracellular trap formation was much greater than that in complement and coagulation cascades, and the proteins of complement and coagulation cascades changed irregularly, while the levels of genes and proteins in the pathway of neutrophil extracellular trap formation were changed consistent with the state of sleep.

Venny analysis showed a total 175 DE genes and proteins that overlapped after sleep deprivation (Figure 3D), and the heat map also showed that shared proteins and genes were respectively mostly downregulated and reduced after sleep deprivation, while the expression of genes and content of proteins was similar on Day2 and Day3 (Figure 4E). Two‐dimensional annotation enrichment analysis, used here to find the commonality of transcriptome and proteome, showed the logFCprotein and logFCgene ranged from −1 to 1 corresponding to the enrichment scores at the protein and transcript levels, respectively. Positive values represent the activation of the process or pathway, and negative values correspond to suppression of the process or pathway. From the map, the common activated biological processes were also enriched in neutrophil degranulation, and neutrophil activation involved in immune response, while the common suppressed biological processes included hemostasis, platelet degranulation, platelet activation, among other. Genes and proteins involved in neutrophil‐related process accounted for major proportion of both (Figure 4F). Taken together, these results suggested that neutrophil‐mediated immune processes best reflect the immune system‐related changes seen after sleep deprivation. By integrating proteomic and transcriptomic, sleep deprivation‐induced prominent immune changes in neutrophil‐mediated immunity, which have a high degree of consistency in the transcriptional level and protein level.

### 2.5 Sleep deprivation resulted in abnormal expression of immune cells and inflammatory factors

**FIGURE 5:**
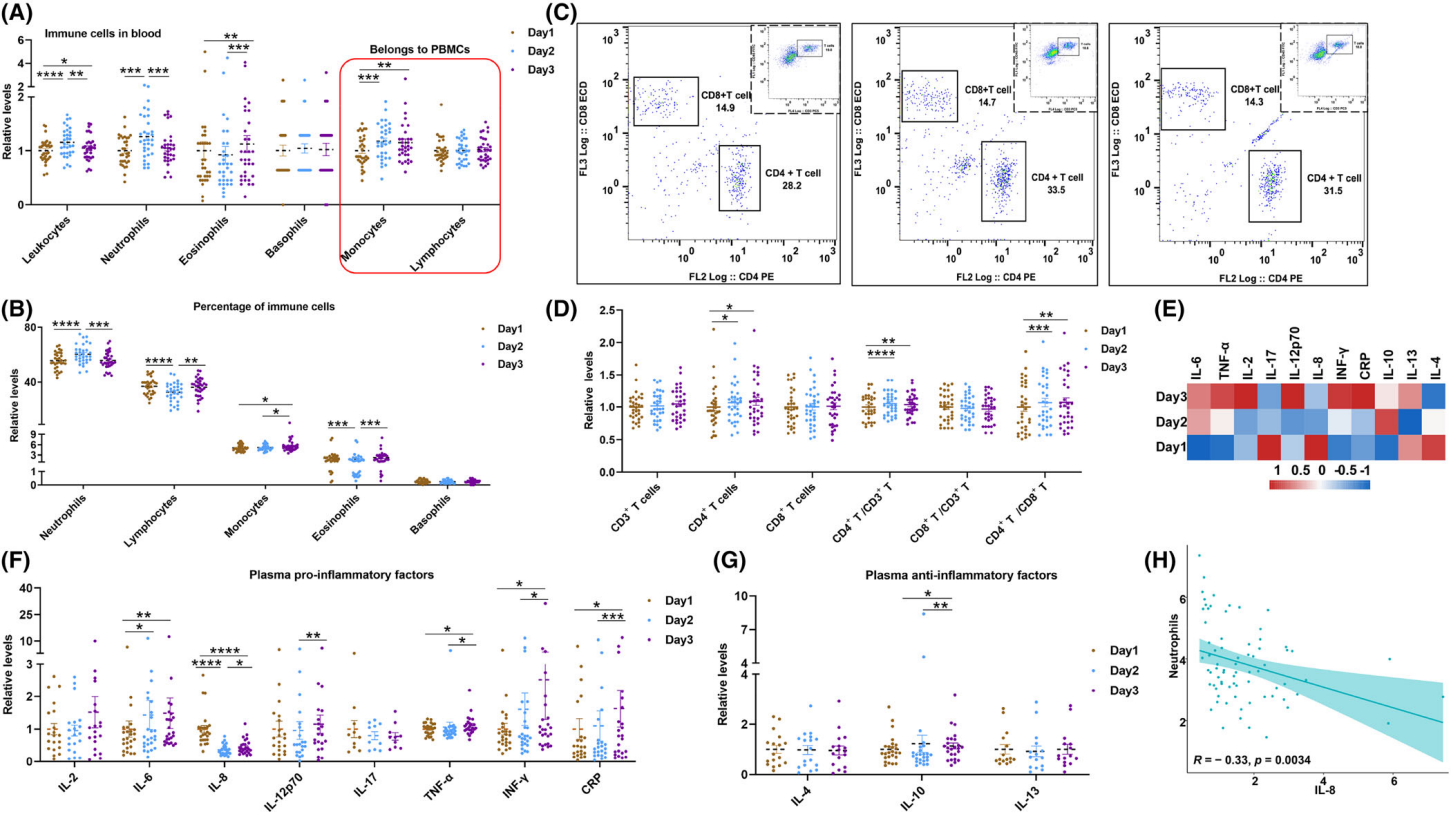
**Sleep deprivation‐resulting changes of immune cell and inflammatory cytokine profiles**. (A) The statistics of immune cells (leukocytes, neutrophils, lymphocytes, monocytes, eosinophils, basophils) from routine blood detection after sleep deprivation, and (B) the proportions of various immune cells in total leukocytes. (C) Representative graphs of flow lymphocyte typing after sleep deprivation, and (D) statistics of the numbers and proportions of CD3^+^T, CD4^+^T, and CD8^+^T cells. (E) Heat map of inflammatory factor profiles after sleep deprivation. (F) Statistical analysis of pro‐inflammatory factors (interleukin [IL]‐2, IL‐6, IL‐8, IL‐12p70, IL‐17, TNF‐α, INF‐γ), c‐reactive protein (CRP) acute response protein and (G) anti‐inflammatory factors (IL‐4, IL‐10, IL‐13). (H) Correction analysis of IL‐8 and neutrophils. Data are presented as the mean ± SEM. *p < 0.05; **p < 0.01; ***p < 0.001; ****p < 0.0001; 15−32 individuals per group.

Immune cells, immune cell typing, inflammatory cytokines, and acute CRP expression were analyzed to determine if the aforementioned effects of sleep deprivation on immune system status could be confirmed. Routine blood results showed the numbers of red blood cells, white blood cells, neutrophils and monocytes were significantly changed after sleep deprivation. Only the number of neutrophils increased significantly on day 2 and decreased significantly on day 3, while there was no difference in their numbers between day 1 and day 3. White blood cells and monocytes were significantly increased in number on day 2, and still significantly increased on day 3, while eosinophils only increased significantly on day 3 (Figure 5A); this result showed that neutrophils may better reflect the effects of sleep changes on immunocompetence. After the proportion calculation, neutrophils, lymphocytes, monocytes, and eosinophils were significantly changed, among which the proportions of neutrophils and lymphocytes changed most obviously (Figure 5B). Since there was no statistical difference between the number of lymphocytes, while significant changes were evident in the proportion of lymphocytes; immunotyping of lymphocytes was performed to identify specific immune cells. Flow cytometric T cell typing showed that CD4^+^T cells increased significantly after sleep deprivation (day 2) and sleep recovery (day 3), and the ratio of CD4^+^T/CD3^+^T and CD4^+^T/CD8^+^T also increased significantly (Figure 5C,D), suggesting that CD4 helper T cells exerted an effective role after sleep deprivation. Therefore, we also detected plasma inflammatory factors, and the heat map showed that most of the anti‐inflammatory and pro‐inflammatory factors were significantly increased on sleep recovery (day 3) (Figure 5E,G), while only IL‐6 was significantly increased on day 2, IL‐8 (accounting for largest change) was significantly decreased on day 2 and day 3. The plasma level of CRP was also increased on day 3, which was consistent with the result of proteomic analysis, a finding that confirmed the reliability of the multiomics results. The altered expression of inflammatory factors and CRP indicated that sleep deprivation induced a delayed effect in the stage of immune status effect, such that most inflammatory factors appeared to be significantly elevated on day 3 after sleep deprivation.

Il‐8 was the only inflammatory factor that was significantly decreased after sleep deprivation (day 2), and this remained significantly decreased after sleep recovery (day 3). IL‐8 chemotaxis and activates neutrophils, promotes lysosomal enzyme activity and phagocytosis of neutrophils, while the number of neutrophils increased significantly after sleep deprivation and the level of IL‐8 significantly decreased, which indicated that elevated neutrophils consumed large amounts of IL‐8. Moreover, the number of neutrophils was significantly negatively correlated with the level of IL‐8 (Figure 5H). Therefore, the result showed that neutrophil elevation required IL‐8 activation, which then depleted amounts of IL‐8 in blood. Sleep deprivation‐induced neutrophil hyperplasia was the initial step, followed by downregulation of related gene and reduced protein expression to inhibit neutrophil activation. The above data show a high concordance between inflammatory factor levels and immune cell counts, and those changes proofed the result of multiomics analysis that sleep deprivation induced the prominent changes in immunity.

### 2.6 SOD1 and *S100A8* may serve as biomarkers of immune disorders caused by sleep deprivation

**FIGURE 6:**
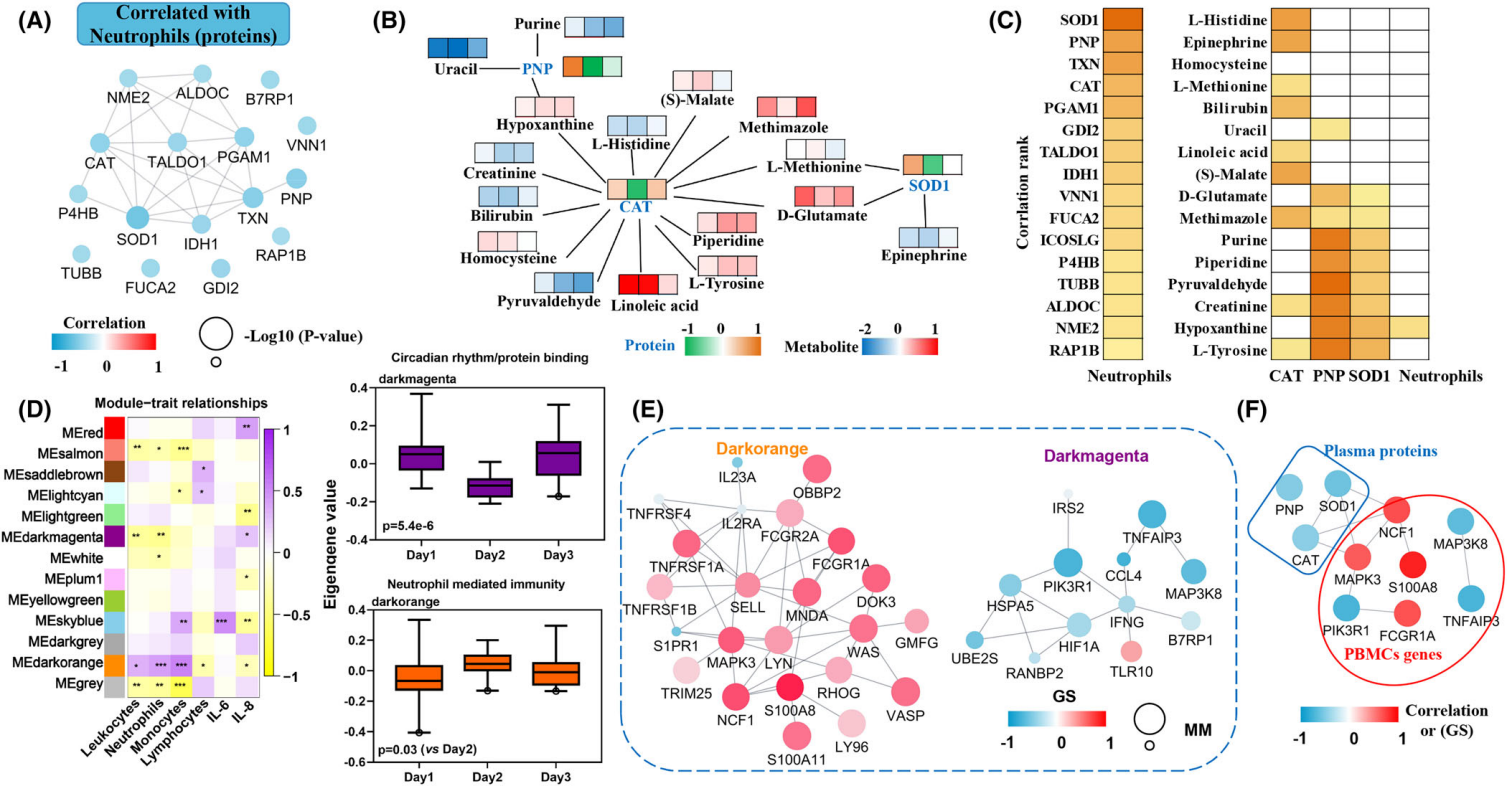
**Correlation molecular network of differential genes and proteins with neutrophils**. (A) String analysis of proteins correlated with neutrophils. Blue represents a negative correlation, and the size of the circle represents the magnitude of the correlation p‐value. (B) Interaction analysis of correlated proteins with total differential metabolites. (C) Correlation rank of proteins correlated with neutrophils, and the correlation of interacted metabolites with correlated proteins and neutrophils. Darker colors represent higher correlations. (D) Weighted gene co‐expression network analysis weighted correlation network analysis (WGCNA) of peripheral blood mononuclear cell (PBMC) gene modules associated with differential immune indices. Modules (*p* < 0.01) that correlated with neutrophils are shown, and the biological processes are listed on the top of module. (E) The network of different modules that correlated with neutrophils. GS represents the correlation between genes and neutrophils, MM represents the weight of the gene in its module. (F) The interaction analysis of correlated proteins with hub genes. The color represents the magnitude of the correlation or GS.

Preliminary analysis showed that biological processes and signaling pathways related to neutrophil‐mediated immune function were dominant after sleep deprivation. Therefore, correlation analysis was used to find the molecular network that regulated the neutrophil changes to reflect disordered immune functions after sleep deprivation. Immune‐related molecules in differential plasma proteins were screened for correlation analysis with neutrophils; the results showed that the expression of differential proteins was negatively correlated with the number of neutrophils, and the network showed that most correlated proteins interacted with each other (Figure 6A). The protein‐metabolite interaction analysis showed that CAT, SOD1, and PNP interacted with differential metabolites, while catalase (CAT) interacted with many metabolites (Figure 6B). The correlation rank showed that superoxide dismutase‐1 (SOD1) occupied the top rank of correlation with neutrophils, and the interacted metabolites were basically correlated with CAT, SOD1, or purine nucleoside phosphorylase (PNP), while hypoxanthine also correlated with neutrophils (Figure 6C). In addition, we analyzed the correlation of proteins with another indicator of neutrophil activation, namely IL‐8, and found that 10 DE proteins significantly correlated with both neutrophils and IL‐8 level, while protein‐protein interaction analysis found that SOD1 was at the center of the protein network (Figure S8A). These results indicated that SOD1 was a central plasma biomarker of the neutrophil‐mediated immune changes following sleep deprivation.

At the PBMC transcript level, the transcriptome weighted correlation network analysis showed that neutrophils were significantly negatively correlated with darkmagenta module (involved in circadian rhythm/protein binding) and positively correlated with darkorange module (mainly focused in neutrophil‐mediated immunity) (*p* < 0.01) (Figure 6D). In the modular gene network analysis, the hub genes (filtered according to the value of the gene significance (GS) and module membership (MM)) positively correlated with the neutrophils included *NCF1, S100A8, MAPK3, FCGR1A*, while the negatively correlated hub genes were mainly *PIK3R1, TNFAIP3, MAP3K8* (Figure 6E). The neutrophil solute factor 1 (*NCF1*) is involved in reactive oxygen species and required for activation of the latent NADPH oxidase. *S100A8* induces chemotaxis and adhesion of neutrophils. *MAPK3* belongs to the MAP kinase signaling pathway, which controls cell proliferation and differentiation. *FCGR1A* regulates the immune response. *MAP3K8* is involved in the production of TNF‐α (tumor necrosis factor‐α) in the inflammatory response. *PIK3R1* participates in phosphatidylinositol 3‐kinase signaling, while *TNFAIP3* is involved in regulating inflammatory responses. The protein function showed that *S100A8* was closely related neutrophil activation, and the GS for neutrophils was prominent among those hub genes. Although the protein level of *S100A8* had no correlation with neutrophil number, the gene level of *S100A8* in PBMCs was significantly correlated with neutrophil content (Figure S8B). Moreover, protein‐gene interaction analysis found that SOD1 interacted with the PBMC gene network, which included *S100A8* (Figure 6F), and the protein expression of S100A8 was also changed in plasma.

In conclusion, the *S100A8* gene level of PBMC may better reflect changes in neutrophil content. At the plasma level, SOD1 may reflect both neutrophils and IL‐8 levels after sleep deprivation. Thus, *S100A8* and SOD1 may act as biomarkers of immune‐function changes associated with sleep deprivation.

### 2.7 Sleep deprivation can also induce enrichment of neurodegenerative disease

Other than the main effects of sleep deprivation on immune‐system function, plasma proteins associated with neurodegenerative diseases (APP, NOTCH3, SNCA, SOD1) were decreased after sleep deprivation, while only ATF6 continued to increase (Figure S9A–C). Interaction analysis of differential genes, proteins, and metabolites with diseases was performed with MetaboAnalyst software to test for potential relationships between sleep deprivation and diseases. The results showed that differential proteins and metabolites were enriched in Alzheimer's disease (AD) and schizophrenia by proteomics analyses on day 2 and day 3, and differential genes and metabolites were also enriched in AD and schizophrenia in the transcriptome (Figure S9D,E). These results indicated that sleep deprivation‐induced changes in peripheral blood multiomics molecules have potential associations with neurodegenerative diseases.

**FIGURE 7:**
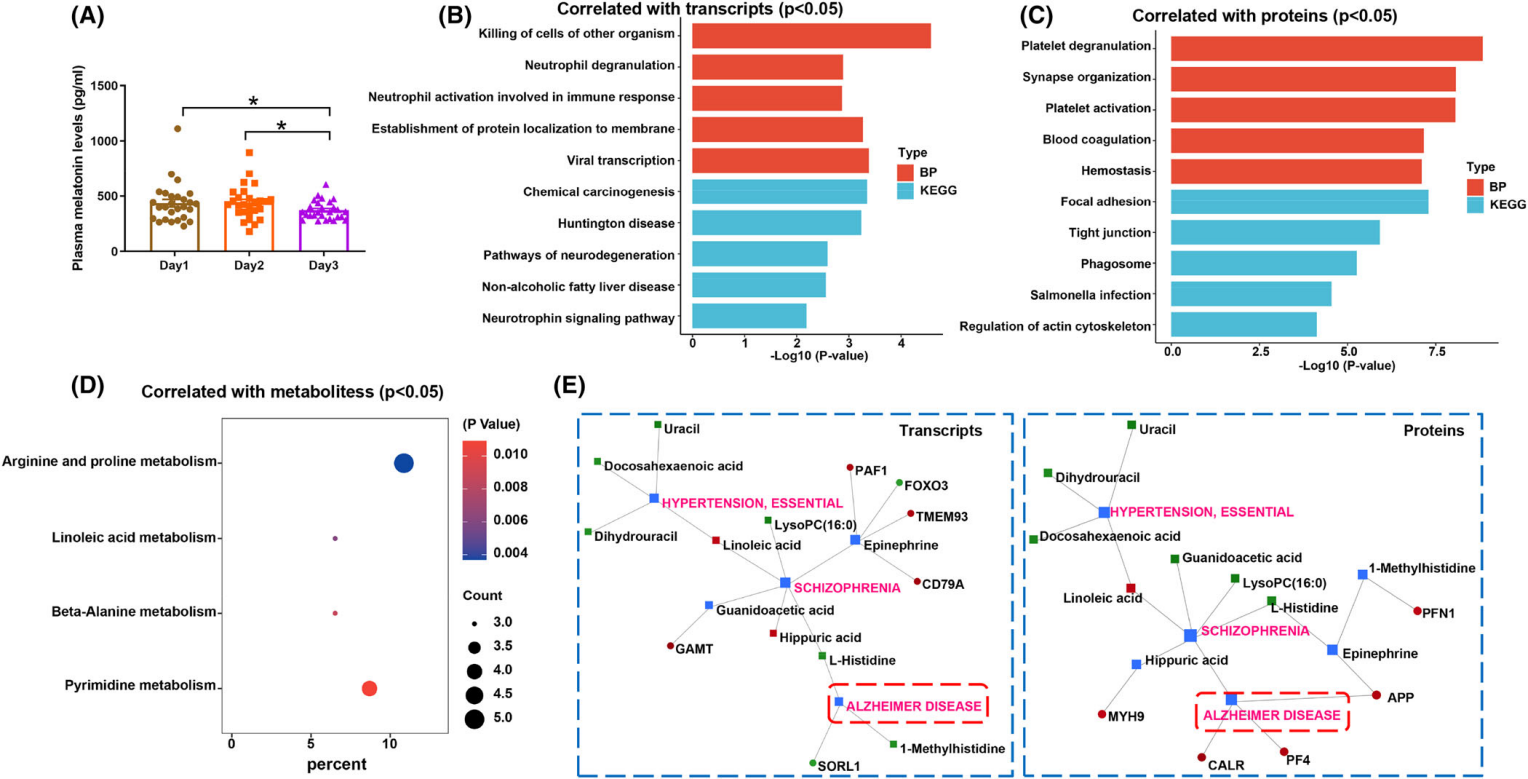
**Analysis of melatonin level and its correlation with multiple‐omics molecules after sleep deprivation**. (A) The melatonin level of plasma after sleep deprivation detected by ELISA kit. Pearson correlation analysis to identify multiomics molecules correlated with melatonin levels, (B) GO and KEGG enrichment analysis of correlated differentially expressed genes, and (C) differential proteins. (D) Pathway analysis of corrected differential metabolites. (E) The interactions analysis of multiomics molecules and disease connected with melatonin. Data are presented as mean ± SEM. *p < 0.05, *n* = 25 individuals per group.

Since melatonin is known to regulate sleep and its secretion synchronizes with circadian rhythms, we assessed melatonin levels after sleep deprivation. There was a significant decrease in plasma melatonin levels on day 3 after sleep deprivation (Figure 7A), suggesting that this can affect the normal secretion of melatonin. Pearson correlation analysis was used to find the differential molecules that correlated with melatonin expression levels. The correlated differential genes were involved in neutrophil degranulation neutrophil activation involved in immune response, Huntington's disease, pathway of neurodegenerative diseases, and neurotrophic factor pathway, among others (Figure 7B). Correlated differential proteins were focused on platelet degranulation, platelet activation, synaptic organization, coagulation, focal adhesion, phagosome, and so on (Figure 7C). Similarly, metabolites that correlated with melatonin were mainly involved in arginine and proline metabolism (Figure 7D). GO and KEGG analysis revealed that molecules correlated with melatonin were also involved in immune and neurodegenerative diseases. Further analysis of disease interaction among metabolites, genes or proteins indicated that melatonin‐correlated omics molecules were associated with hypertension, schizophrenia and AD. Therefore, these results indicated that the changes of melatonin caused by sleep deprivation have a potential association with neuropsychiatric and neurodegenerative diseases (especially AD). In addition to the effects of immune function, sleep deprivation also indued a widely enrichment pathways of neurodegenerative diseases and decreased melatonin level in the plasma, these changes implied that sleep deprivation may contribute to the potential development of neurodegenerative diseases.

## 3 DISCUSSION

This is the first time that an integrated multiomics approach has been used to assess the impact of sleep deprivation on blood parameters. Transcriptome and proteome analysis showed that sleep deprivation was associated with genomic downregulation and reduction of many plasma proteins, changes that were not fully rectified during the 1–2‐day recovery period. Bioinformatics analysis showed that the immune system is the most sensitive to sleep deprivation. This is consistent with previous research demonstrating that sleep deprivation can adversely impact immune function and that brief sleep recovery does not restore normal immune status.^28^ Metabolomic analysis showed that differential metabolites were mainly involved in amino acid metabolism and carbon metabolism, among which arginine and proline metabolism and the TCA cycle were the main processes. And the analysis also indicated that the immune system may be somewhat suppressed after sleep deprivation, a finding consistent with the results of proteomic analysis. Moreover, combined analysis of differential proteins and metabolites showed that innate immunity and immune system processes were the principal functions perturbed by sleep deprivation.

Analysis of the peripheral blood mononuclear cell transcriptome showed that sleep deprivation resulted in downregulation of a large number of immune‐related genes that remained downregulated upon sleep recovery. Correction analysis of DE genes and proteins showed that neutrophil‐ and complement‐mediated immune processes accounted for the major changes induced by short‐term sleep deprivation. Compared with the complement‐involved signaling pathway, sleep deprivation significantly changed the expression of genes in the neutrophil‐related signaling pathway and had a greater overlap with the DE proteins. Blood analysis also reflected altered immune status following sleep deprivation: leukocytes, neutrophils, monocytes, and eosinophils were increased, and the proportion of each cell type was changed significantly. Plasma interleukin‐8 (IL‐8), which plays a pathogenic role in acute inflammation by recruiting and activating neutrophils,^29^ was significantly decreased in both sleep deprivation and recovery states. By correlation analysis, plasma superoxide dismutase‐1 (SOD1) was most likely to be involved in changes in neutrophil‐mediated immunity after sleep deprivation, followed by catalase (CAT), PNP, and hypoxanthine. *S100A8*, inducing neutrophil chemotaxis and adhesion^30^ and serving as a biomarker for inducing neutrophil activation,^31^ was the crucial hub gene connected with neutrophils, which revealed through correlation analysis of neutrophils and IL‐8 on the PBMC transcriptome. Thus, *S100A8* in PBMC and SOD1 in plasma appear from the present results to be sensitive immune‐system biomarkers of sleep changes at gene and protein levels, respectively. By multiomics analysis, we found that sleep deprivation induced levels of multitudinous molecules change. Among them, the reduction of levels of molecules involving immune function (especially neutrophil‐mediated immune processes) was most obvious. Levels of molecules involving in processes such as neutrophil‐mediated immunity and cholesterol metabolism decreased by sleep deprivation and recovered after sleep recovery, whereas the sleep deprivation‐induced decrease level of molecules relate to complement and coagulation cascades and gluconeogenic processes could not be reversed by sleep recovery.

Moreover, disease enrichment analysis showed that sleep deprivation led to a substantial enrichment of categories of neurodegenerative disease, including AD, PD (Parkinson‘s disease), and ALS (Amyotrophic lateral sclerosis), which implied sleep deprivation or loss has been linked to the potential for neurodegenerative diseases, such as memory loss and cognitive impairment^32^, ^33^ and increased brain Aβ.^9^ In addition, we further analyzed plasma proteins that did not change during sleep deprivation and found that there have many immune molecules/signaling pathways that are not affected by transient sleep deprivation. Notably, in this study, although the integration analysis of PBMC transcriptome, plasma proteome, and plasma metabolome has found a lot of changes after sleep deprivation, these changes may be caused by different cells at different times.

However, there also have some limitations in this research. Firstly, the immune analysis of PBMC was not detailed enough. Secondly, lipidomics analyses of plasma metabolites were not performed. Finally, the major limitation is that the changes of multiomics molecules and/or immune markers were not detected during a longer recovery period after sleep deprivation. Considering the above‐mentioned limitations in the experimental design, although this study cannot determine whether the harms of sleep deprivation are restored or persisted in the longer future, at least demonstrated that even one day's sleep deprivation, such as staying up late or shift work in daily life, has enormous potential health risks. Moreover, those omics data also indicated that sleep deprivation has a greater impact on peripheral blood molecules, which may be a beneficial method for monitoring sleep deprivation‐induced disease by peripheral blood. And the long‐term follow‐up of these data in the future will be helpful to explore the molecular basis of the long‐term harm caused by sleep deprivation.

## 4 CONCLUSION

The multiomics analysis of blood from healthy young male and female adults showed that short‐term sleep deprivation principally perturbs immune function and neutrophil activation dominated sleep deprivation‐induced changes in immune status. The level of SOD1 plasma protein level and transcriptional profile of *S100A8* in PBMC may serve as biomarkers reflecting changes in immune status caused by sleep deprivation.

## 5 MATERIAL AND METHODS

### 5.1 Participants and study design

We recruited 32 medically examined healthy Chinese volunteers to participate in the sleep‐deprivation experiments. Study participants comprised 14 males and 18 females aged 22−27 years (mean ± s.d. 24.2 ± 1.4 years) and with a body mass index of 16.8–30.3 (mean ± s.d. 22.1 ± 3.4). All participants had no significant medical history or chronic disease. Blood (10 ml) draws were taken as follows at 08:30 a.m. (1) following an overnight fast (day 1, baseline blood sample), (2) after 24 h of sleep deprivation (day 2, sleep‐deprivation blood sample) and (3) after a further 24 h during which participants resumed a normal diet and sleep (day 3, recovery blood sample) (Figure 1A). All participants underwent rigorous physical screening, consistent schedules and diets to avoid experimental errors in baseline data. Heart rate and blood pressure was recorded prior to each blood collection.

### 5.2 Biochemical detection and blood routine test

Daily blood samples were analyzed by the Shenzhen Occupational Disease Prevention Hospital for content of red blood cells, white blood cell content and platelets, glucose and lipid content, liver and kidney function, and HbAc1.

### 5.3 Transcriptomics analysis of PBMCs

PBMCs were isolated with lymphocyte separation solution (Ficoll, USA) followed by total RNA extraction with the RNeasy Mini Kit (Qiagen, China), and a total 60 samples from 20 participants were used for transcriptomics analysis. This protocol was referred to previous studies.^34^ mRNA was obtained from total RNA by oligo(dT)‐attached magnetic beads and used for library construction. After cDNA synthesis, end repair and joint connection, with circularization by the splint oligo sequence, the libraries obtained thereby were amplified with phi29 for DNA nanoball (DNB) construction and sequencing using a MGI2000 platform (BGI‐Shenzhen, China). Sequencing data were filtered with SOAPnuke (v1.5.2) to acquire clean reads (stored in Fragments per kilobase of sequence per million mapped reads, i.e., FPKM format) and then mapped to the *Homo sapiens* genome using HISAT2 (v2.0.4). Bowtie2 (v2.2.5) aligned the clean reads to the reference gene sequence, after which RSEM (v1.2.12) was used to calculate the gene expression level of each sample. Differential expression analysis was performed using DESeq2(v1.4.5) with *Q* value < 0.05.

### 5.4 Proteomics analysis of plasma

Supernatant plasma was removed after centrifuging (3000 g) the EDTA‐treated blood sample, a total 90 samples from 30 participants were used for proteomics analysis. High Select Top14 Abundant Protein Depletion Mini Spin Columns (Thermo Fisher, USA) were used to remove the top 14 high‐abundant proteins (albumin, IgA, IgD, IgE, IgG, IgG [light chains], IgM, etc.) from plasma samples, after which the solvent was replaced by urea lysis buffer (8 M urea, 1 × PBS, 1xProtease and Phosphatase Inhibitor, PH = 8.0) by ultrafiltration tube. The protocol used for proteomic analysis has been described previously.^35^ In brief, protein concentration was measured by BCA kit (Thermo Fisher, USA) and then 50 μg protein were incubated with dithiothreitol (DTT) followed by incubation with indole‐3‐acetic acid, and trypsin digestion (Promega, USA). After digestion, the peptide was desalted and redissolved with trimethylammonium bicarbonate. The TMT10plex kit (Thermo Fisher, USA) was used to label peptides, which were then desalted and fractionated by UltiMate 3000 UHPLC for a total of 15 fractions. Finally, the fractions were analyzed by LC‐MS/MS (Q‐Exactive, Thermo Fisher, USA). Proteome Discoverer Software (2.5) was used for protein quantification and annotation from the database of UniProt‐*Homo sapiens* (2021 update). Samples in which protein expression had a coefficient of variation < 0.3 were retained, and the blank value (<0.6 proportion) was filled using a random forest algorithm. The guideline for protein differentiation was set at *q* < 0.05 (Bonferroni‐Holm correction) using Perseus (1.5) software.

### 5.5 Metabolomics analysis of plasma

Protein metabolites were extracted from plasma by precipitation with cold methanol,^36^ a total 96 samples from 32 participants were used for metabolomics analysis. The metabolites were lyophilized, redissolved in 5% methanol, and analyzed by LC‐MS/MS (liquid chromatography‐tandem mass spectrometry) using reverse phase chromatography to acquire data in positive and negative ion modes. Data were analyzed using Compound Discoverer 3.0 for retention time alignment, peak extraction and area normalization, and metabolite identification. Inter‐ and intra‐batch variation was corrected by robust LOESS (locally estimated scatterplot smoothing) signal based on QC (quality control) samples. The DE metabolite detection was set at *q* < 0.05 (Bonferroni‐Holm correction), and fold change >1.1. DE metabolites were matched by ID using the KEGG metabolic database to facilitate functional analysis.

### 5.6 Bioinformatics analysis

PCA (principal component analysis) was performed to identify differences in multiomics results before and after sleep deprivation. For DE genes and proteins, the Cluster Profiler plugin of R studio was used to perform enrichment analysis with GO and KEGG, and the Mfuzz plugin was used to perform temporal cluster analysis. Signaling pathways were drawn using Clue GO to display DE proteins. The interaction of proteins and metabolites was analyzed by MetaboAnalyst 5.0 software. The correlation between DE molecules and biochemical criteria was analyzed using the Hmisc plugin in R studio, and WGNA was used to discover molecular modules related to phenotypic changes. Differential metabolites were enriched for KEGG analysis, and correlation analysis of proteomics and metabolomics used the “Wu Kong” platform (https://www.omicsolution.com).

### 5.7 Flow T cell typing analysis

Lymphocytes were separated from fresh blood by Ficoll buffer and resuspended in stain buffer. After washing twice in cold stain buffer, lymphocytes were diluted for a final concentration to 2 × 10^7^ cells/ml. Appropriate amounts of specific surface antibodies (CD3, CD4, CD8) were added to the cell suspension and incubated on ice in the dark. After incubation, cells were washed twice with stain buffer and then resuspended in stain buffer and analyzed by flow cytometry.

### 5.8 Inflammatory factor detection

Plasma levels of inflammatory cytokines were measured by MESOTM QuickPlex SQ 120 (Meso Scale Diagnostics, Rockville, MD, USA). Briefly, after the plasma samples were centrifuged for 10 min at 12,000 g, the supernatant was diluted with MSD solution. The standard and samples were added to the MSD plate for incubation, then washed with 1 × PBS and re‐incubated with detection antibody. After incubation, the plate was washed with 1 × PBS and added detection liquid, and then measured by Meso QuickPlex SQ 120.

### 5.9 ELISA (enzyme‐linked immunosorbnent assay) detection for melatonin, CRP, and IL‐17 levels in plasma

Levels of CRP (C‐reactive protein), IL‐17, and melatonin in plasma supernatant were detected according to the protocol for ELISA kits (elabscience, China). In brief, samples were diluted in a suitable range (according to instructions) and then added with the standard to the plate. For CRP and melatonin detection, samples were incubated with biotinylated antibody immediately for a certain period of time (90 min) at optimum temperature (37°C). For IL‐17 detection, the sample was incubated with the plate followed by incubation with the biotinylated antibody. Then, the plate was washed several times and incubated with HRP (horseradish peroxidase) enzyme conjugate working solution, substrate solution, and stop solution. Finally, the plate was immediately read at 450‐nm wavelength by Microplate Reader (Bio‐RAD, USA).

### 5.10 Statistical analysis

All data are presented as mean ± standard error (SEM, standard error of mean). The two‐tailed Student's t‐test and paired Wilcoxon test were used to determine statistical significance between two groups. Statistical plots were performed with GraphPad Prism 8 software.

## AUTHOR CONTRIBUTIONS

CYC and JW drafted the manuscript and analyzed the data. CY, HTY, BGZ, XY, and BCX helped sample collection and processing. YMX, SPL, ZJZ, FQZ, JJL, XFY, and GPL designed the study. XFY and GPL revised the manuscript. All authors have read and approved the final manuscript.

## CONFLICT OF INTEREST STATEMENT

The authors declare no competing interests.

## ETHIC STATEMENT

The study was approved by the Ethics Committee of Shenzhen Center for Disease Control and Prevention (QS2021040014) and China Clinical Trials Registry (ChiCTR2100045085). Participants provided written informed consent for their study participation.

## Supporting information

Supplimentary information

## Data Availability

The data sets used for the current study are available from the corresponding author upon reasonable request.
