# Supplementary material for: Multiomics analysis of human peripheral blood reveals marked molecular profiling changes caused by one night of sleep deprivation
Source: MedComm (2020). 2023 Apr 30;4(3):e252. doi: 10.1002/mco2.252 (PMC10149526; doi:10.1002/mco2.252)
Supplement: Supplementary file 1 — Supplimentary information [file MCO2-4-e252-s001.docx]

**Multi-omics analysis of human peripheral blood reveals marked molecular profiling changes caused by one night of sleep deprivation**

Chongyang Chen^1,2,#^, Jing Wang^1,#^, Chao Yang^4^, Haitao Yu^3^, Bingge Zhang^3^, Xiao Yang^1^, Bocheng Xiong^1^**,** Yongmei Xie^5^, Shupeng Li^6^, Zaijun Zhang^7^, Feiqi Zhu^4^, Jianjun Liu^1^, Gong-Ping Liu ^3,8,*^ and Xifei Yang ^1,*^

**Figure S1-9**


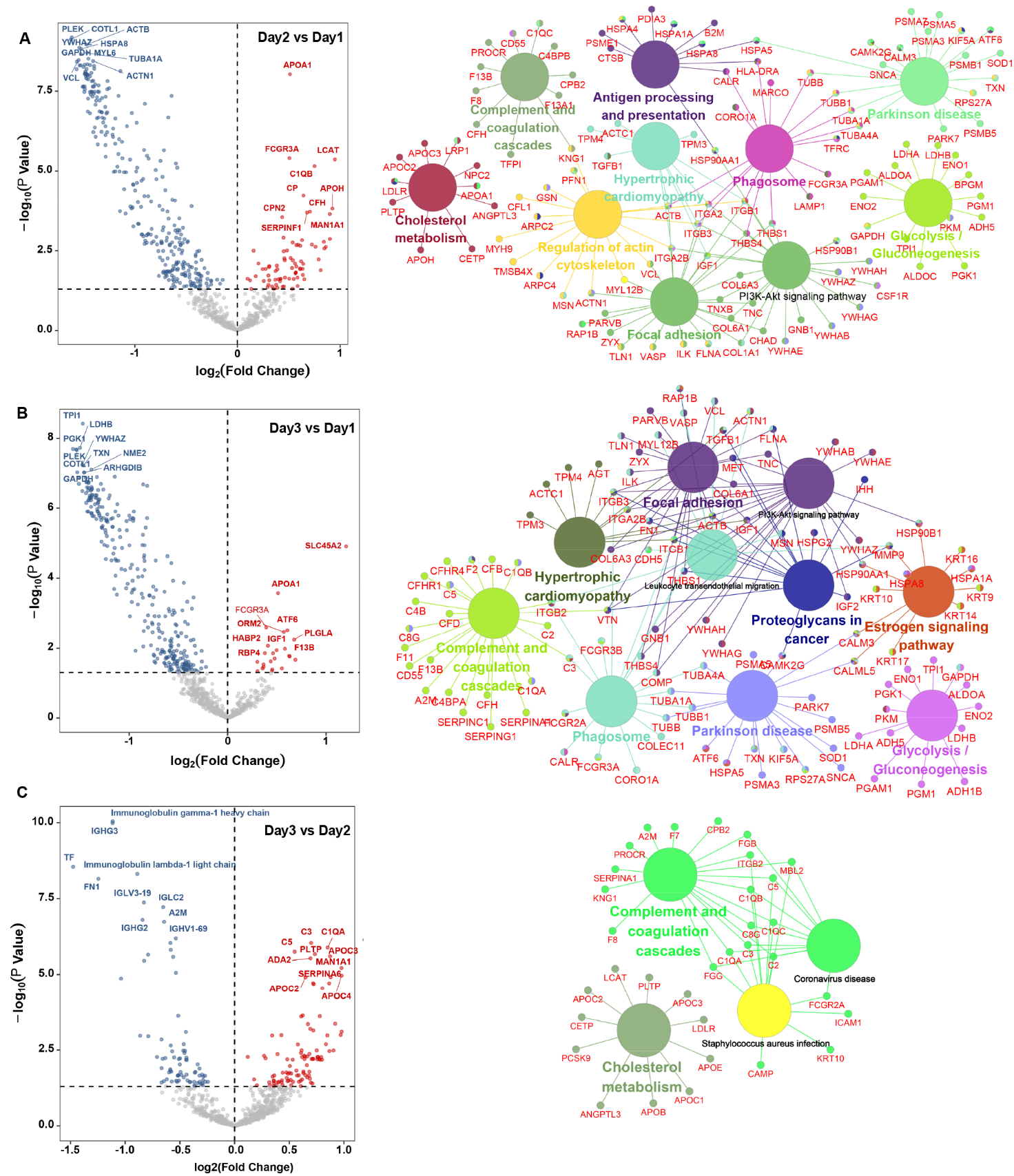


**Figure S1. Differential plasma protein analysis after sleep deprivation.**

Volcano plot and pathway map of differential plasma proteins after sleep deprivation. (A) Volcano plot and pathway map on Day2, and (B) Day3 after sleep deprivation. (C) Volcano plot and pathway map of compared group in Day3 vs Day2. The top 10 differential proteins are shown in Volcano plots, where red represents increased and blue represents decreased presence. Clue GO software was used to visualize pathways shown in different colors, with proteins marked in red. Each signaling pathway contained at least 10 differential proteins.


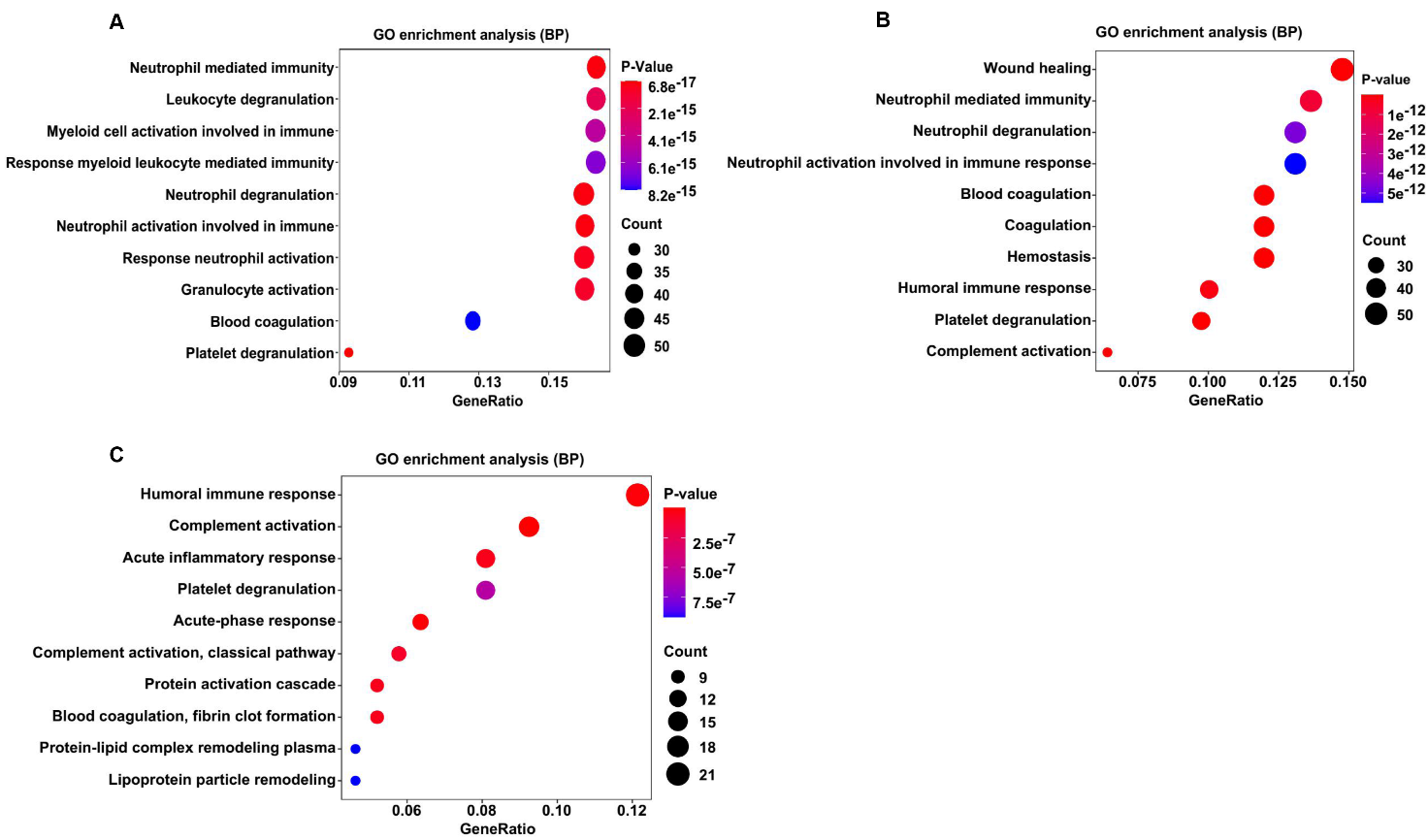


**Figure S2. Gene ontology analysis of differential proteins after sleep deprivation.**

(A) The biological process of differential proteins on Day2, and (B) Day3 after sleep deprivation. (C) The biological process of compared group in Day3 vs Day2. The p value of GO enrichment was corrected with Bonferroni-Holm (BH), and the top 10 biological processes are displayed.


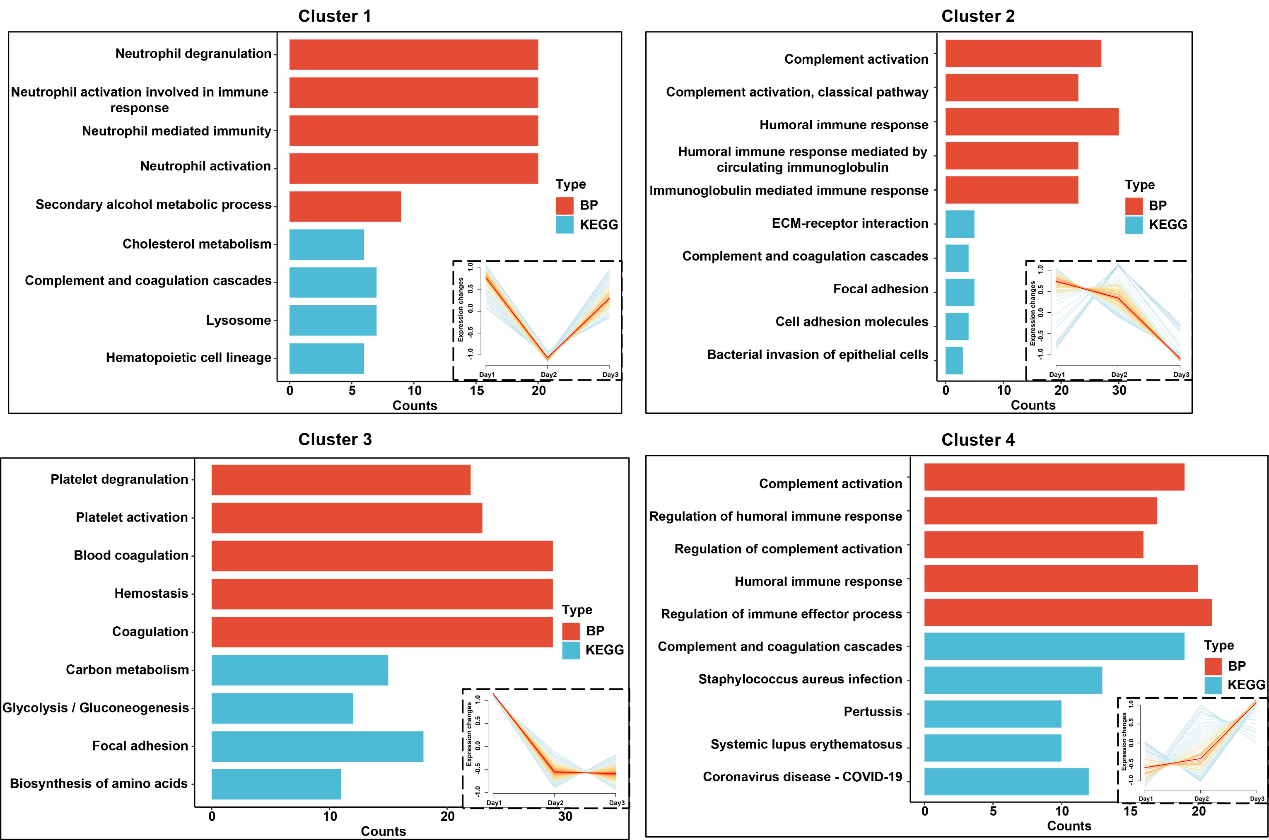


**Figure S3. Temporal cluster analysis of differential plasma proteins after sleep deprivation.**

Temporal clustering of total differential plasma proteins before and after sleep deprivation (Day2 vs Day1 and Day3 vs Day1) was performed using Mfuzz analysis to find cluster modules closely related to sleep changes. GO and KEGG analyses were performed to annotate biological processes and pathways of different clusters. The figure shows the top 5 biological processes and pathways in which clusters were enriched.


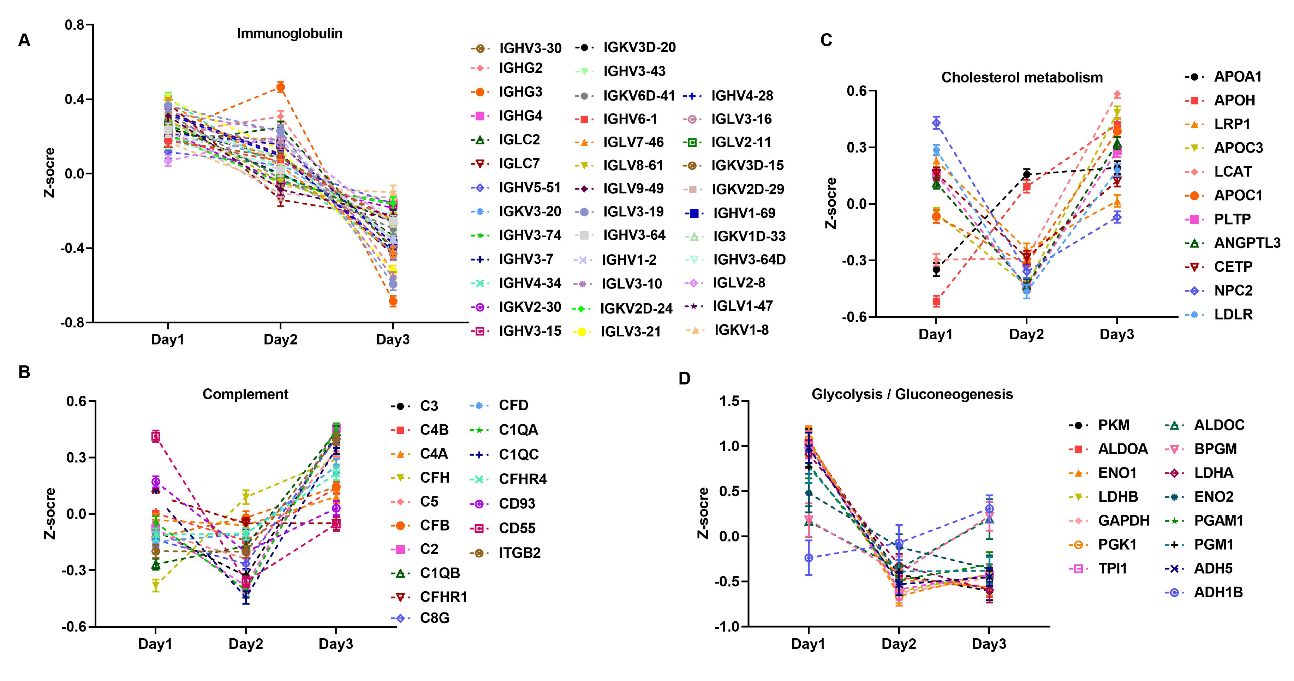


**Figure S4. Characterization of changes in proteins closely related to immunity and metabolism.**

GO analysis to classify immune and metabolic process proteins. (A) Immunoglobulin, (B) complement, (C) cholesterol metabolism, (D) glycolysis and gluconeogenesis. The change of each protein is shown as an average and is connected by dotted lines.


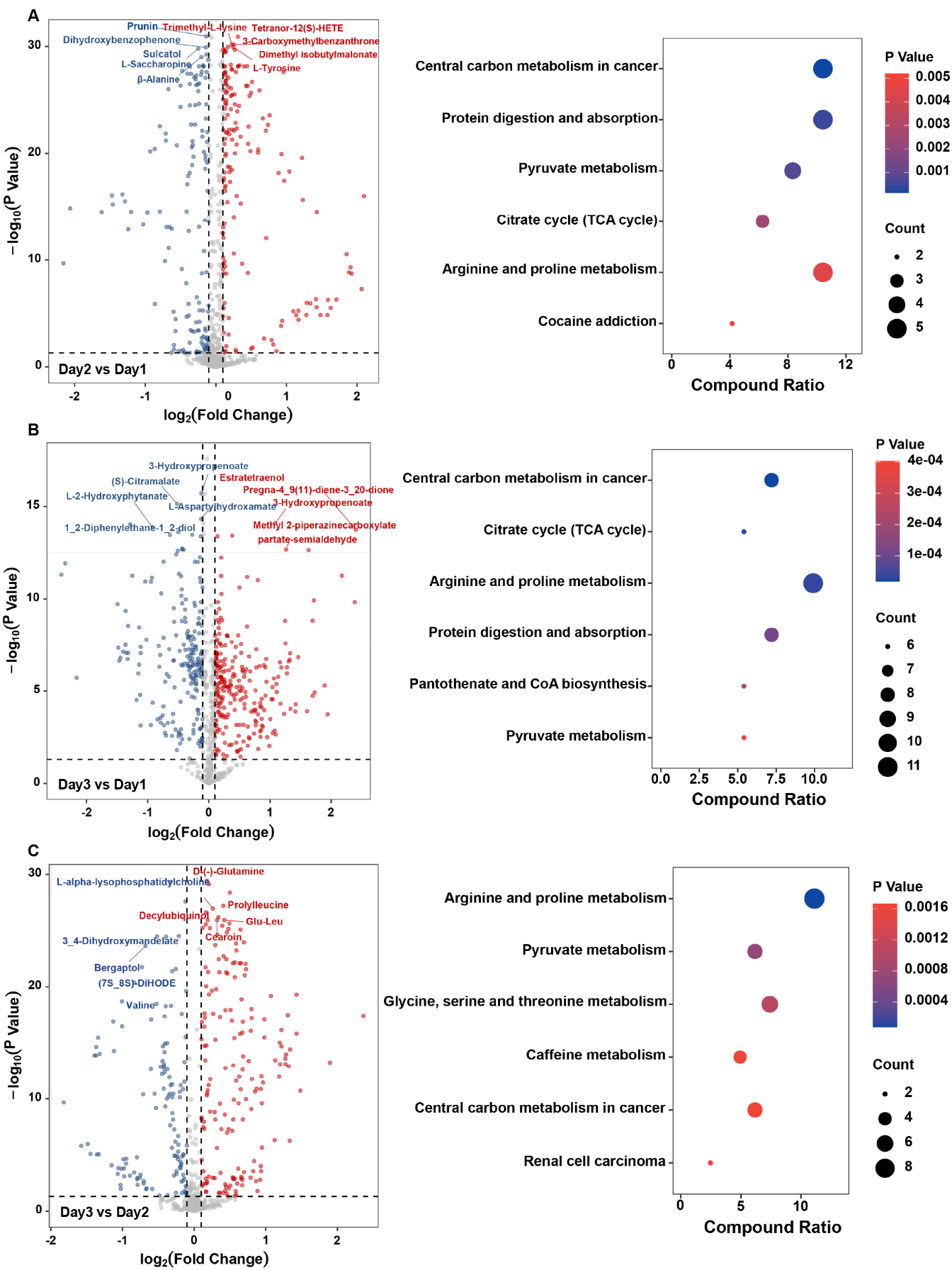


**Figure S5. Basic analysis of plasma metabolites after sleep deprivation.**

Metabolite changes induced by sleep deprivation were studied by plasma metabolomics. The Volcano plot and KEGG metabolic pathway analysis charactered the differential metabolites before and after sleep deprivation. (A) Differential metabolites on Day2 and (B) Day3. (C) Differential metabolites in compared group of Day3 vs Day2. Pathway analysis of metabolites with clear ID of KEGG and HMBD. The top 5 differential metabolites are shown in the Volcano plot, and the top 6 metabolic pathways are presented.


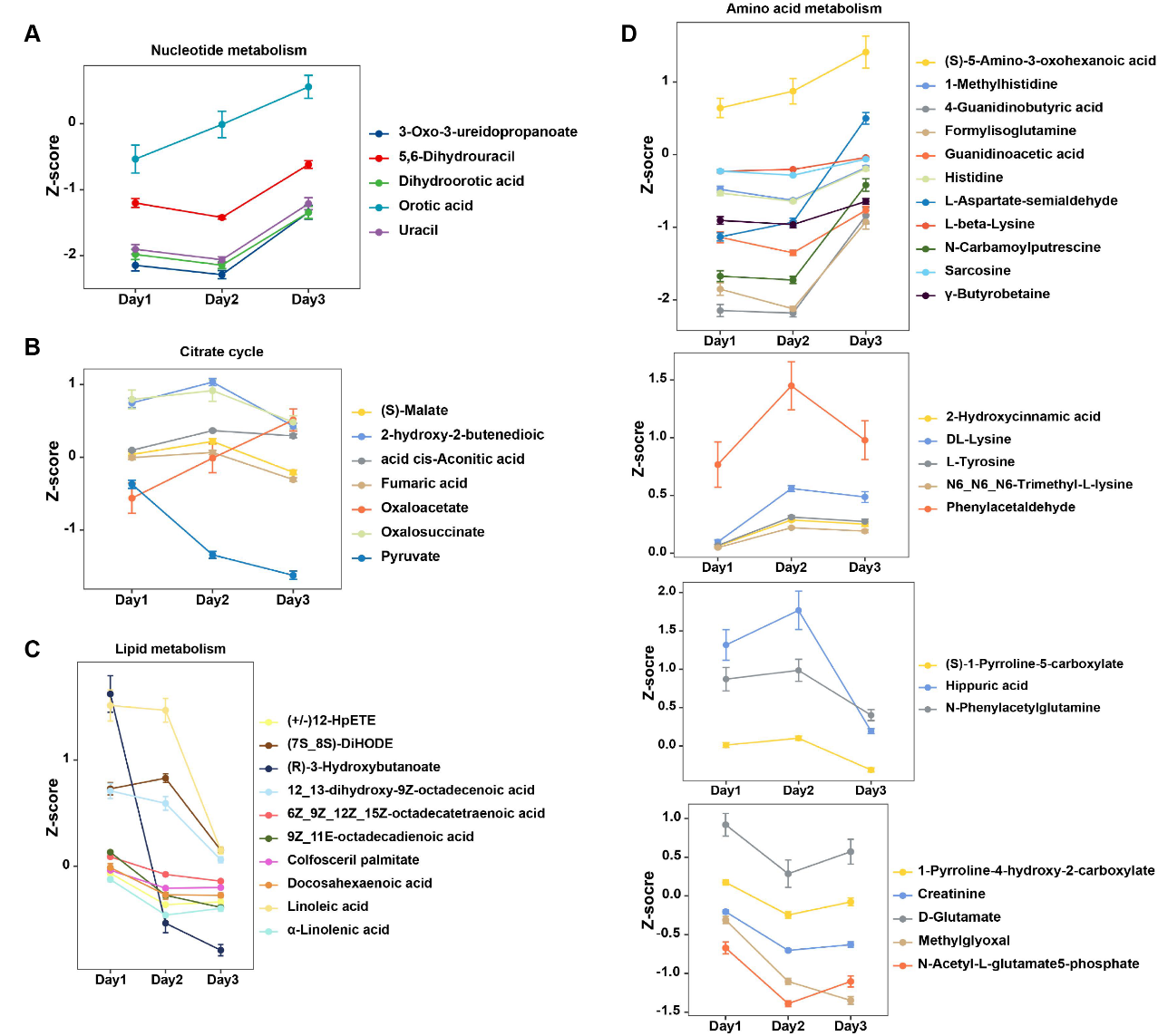


**Figure S6. Expression changes of different types of metabolites before and after sleep deprivation.**

The expression trend of different types of metabolites. (A) The expression of metabolites involved in nucleotide metabolic process, (B) TCA cycle, (C) lipid metabolic process and (D) amino acid metabolism.


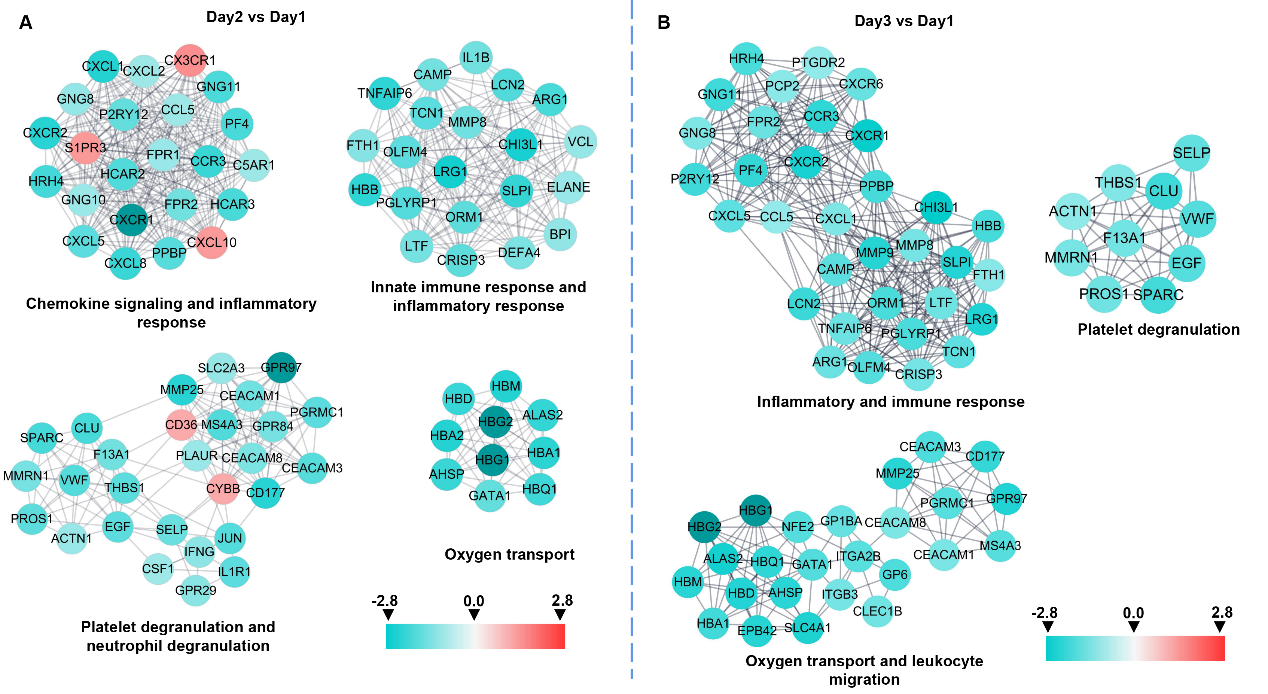


**Figure S7. Molecular complex detection (MCODE) analysis of differential genes after sleep deprivation.**

(A) MCODE analysis of differential genes on Day2 and the hub modules after annotation by GO analysis. (B) MCODE analysis of differential genes on Day3. Red represents up-regulation of gene expression and green represents down-regulation.


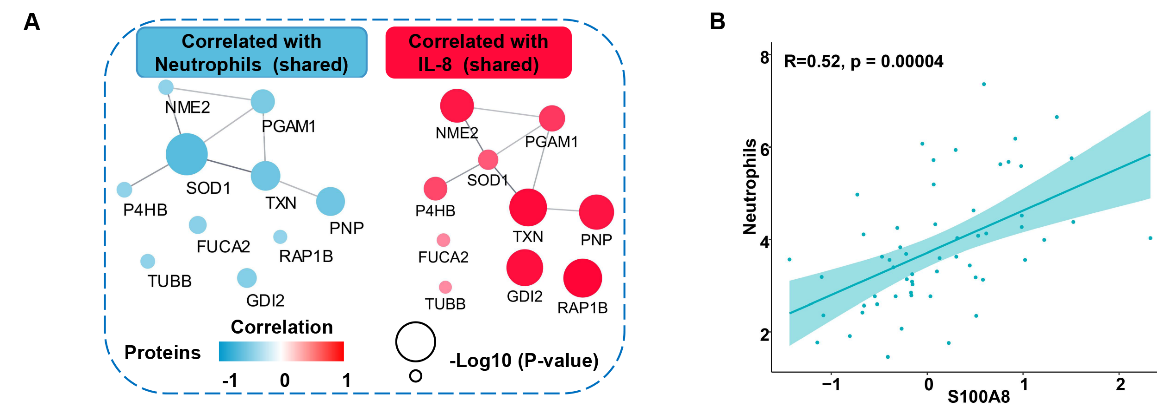


**Figure S8. Correlation analysis of differential molecules with both neutrophils and IL-8.**

(A) Interaction analysis of shared correlated proteins that correlated with neutrophil levels and IL-8. (B) Correlation analysis of S100A8 gene level with neutrophils.


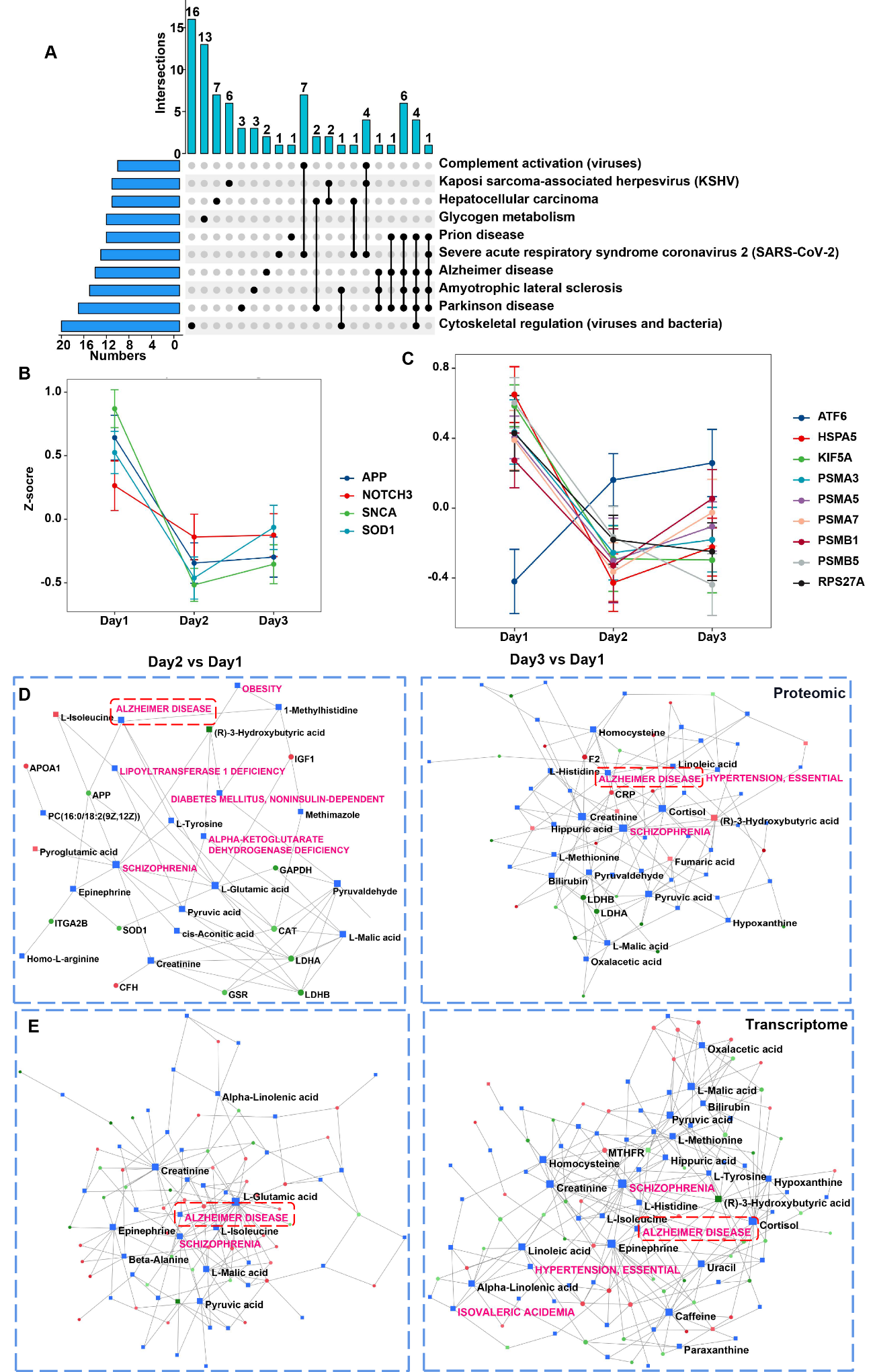


**Figure S9. Disease analysis of multi-omics differential molecules after sleep deprivation.**

(A) The KEGG disease database was used for enrichment analysis of differential proteins. Venn diagrams were used to analyze the number of differential proteins shared in various diseases. (B) The expression of neurodegenerative disease-specific proteins, and (C) shared differential proteins in AD, PD, ALS and prion disease. (D) MetaboAnalyst analysis of the interaction among differential proteins, metabolites and disease, and (E) the interaction among differential genes, metabolites and disease. Red dots represent up-regulated genes or increased proteins, and green dots represent down-regulated genes or reduced proteins. Blue represents metabolites and disease was marked with pink.
